# High-throughput binding characterization of RNA aptamer selections using a microplate-based multiplex microcolumn device

**DOI:** 10.1007/s00216-014-7661-7

**Published:** 2014-02-20

**Authors:** Kylan Szeto, Sarah J. Reinholt, Fabiana M. Duarte, John M. Pagano, Abdullah Ozer, Li Yao, John T. Lis, Harold G. Craighead

**Affiliations:** 1 School of Applied and Engineering Physics, Cornell University, Ithaca, NY 14853 USA; 2 Department of Molecular Biology and Genetics, Cornell University, Ithaca, NY 14853 USA

**Keywords:** Microcolumn, Aptamer, SELEX, Multiplex, High-throughput

## Abstract

We describe a versatile 96-well microplate-based device that utilizes affinity microcolumn chromatography to complement downstream plate-based processing in aptamer selections. This device is reconfigurable and is able to operate in serial and/or parallel mode with up to 96 microcolumns. We demonstrate the utility of this device by simultaneously performing characterizations of target binding using five RNA aptamers and a random library. This was accomplished through 96 total selection tests. Three sets of selections tested the effects of target concentration on aptamer binding compared to the random RNA library using aptamers to the proteins green fluorescent protein (GFP), human heat shock factor 1 (hHSF1), and negative elongation factor E (NELF-E). For all three targets, we found significant effects consistent with steric hindrance with optimum enrichments at predictable target concentrations. In a fourth selection set, we tested the partitioning efficiency and binding specificity of our three proteins’ aptamers, as well as two suspected background binding sequences, to eight targets running serially. The targets included an empty microcolumn, three affinity resins, three specific proteins, and a non-specific protein control. The aptamers showed significant enrichments only on their intended targets. Specifically, the hHSF1 and NELF-E aptamers enriched over 200-fold on their protein targets, and the GFP aptamer enriched 750-fold. By utilizing our device’s plate-based format with other complementary plate-based systems for all downstream biochemical processes and analysis, high-throughput selections, characterizations, and optimization were performed to significantly reduce the time and cost for completing large-scale aptamer selections.

**Electronic supplementary material:**

The online version of this article (doi:10.1007/s00216-014-7661-7) contains supplementary material, which is available to authorized users.

**Figure:**
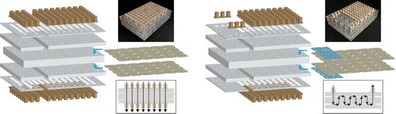
Schematic breakdown of a microplate-based enrichment device for the selection of aptamers (MEDUSA), which can be customized and assembled in both parallel and serial configurations. Up to 96 selections can be performed simultaneously.

## Introduction

Systematic Evolution of Ligands by EXponential enrichment (SELEX) is an in vitro selection method used to generate high affinity ligands for specific target compounds [1–3]. These selected molecules, called aptamers, are derived from large libraries of nucleic acids with random sequences through an iterative process of binding, partitioning, and amplification of sequences that bind to the target. This process enriches the initial random library for higher binding affinity sequences, and the cycle is repeated until the molecules in the enriched pools converge on the highest affinity sequence. Since this method was first introduced, aptamers have become valuable tools in biotechnology, diagnostics, and therapeutics [4].

There is interest in improving SELEX technology to obtain highly specific aptamers much more rapidly. However, despite their potential, many technologies are difficult to scale for multiplexed or parallel selections. For example, Park et al. and Ahn et al. used microfluidic sol–gel devices that could utilize up to five targets for multiplexing [5, 6], but currently, no large-scale microfluidic selections have been demonstrated. Large-scale parallel selections have been done with microplate technologies, which are of particular interest due to the availability of protocols and automated liquid handling devices [7, 8]. However, in contrast to microfluidic devices that utilize flow and other dynamic behavior, most of these selections rely on traditional equilibrium solution binding [9] or interactions with target molecules that are bound or adsorbed to the plate surface [10, 11].

Despite advances toward more sophisticated and automated SELEX, little has been done to characterize and optimize new or current technologies and recent binding studies show significant discrepancies with existing theory [12, 13]. Therefore, empirical methods have been used to optimize selection conditions and aid the development of new models [14]. As new high-throughput technologies emerge, these studies will become even more important in order to obtain the most effective and robust selections under the available parameters.

To address these issues, we have developed a high-throughput device called Microplate-based Enrichment Device Used for the Selection of Aptamers (MEDUSA). This device is designed around a 96-well microplate format, which not only allows for high-throughput selections, but also complements existing plate-based methods and technologies for sample handling and has the potential for automation. MEDUSA is a substantial expansion of our previously reported modular and multiplexable microcolumns, which achieve non-equilibrium selections by utilizing dynamic flow rates shown to optimize the enrichment of aptamers [13]. We demonstrate the use of MEDUSA by performing 96 simultaneous selection tests to characterize the binding of a number of RNA aptamers against various targets. In total, the characterization tests performed on MEDUSA shed light on the critical binding behaviors of specific and background binding aptamers that fundamentally limit the performance and sensitivity of solid-phase affinity selections.

## Materials and methods

### Protein immobilization on affinity resins

Nickel-nitrilotriacetic acid (Ni-NTA) Superflow or glutathione-agarose (GSH) resins were extensively washed with binding buffer [10-mM N-2-hydroxyethylpiperazine-N′-ethanesulfonic acid − KOH pH 7.6, 125-mM NaCl, 25-mM KCl, 5-mM MgCl_2_, and 0.02 % Tween-20]. Hexahistidine- or GST-tagged proteins (see Table S1) were immobilized at the desired concentrations onto the washed resin in 10 % slurry with binding buffer and incubated at 4 °C with constant mixing for 1 h.

### RNA library and aptamers

The random RNA library used in the experiments, hereafter referred to as N70 library, contains ∼5 × 10^15^ sequences of 120-nucleotide (nt) RNA molecules and was prepared as described previously [13]. This library consists of a 70-nt random region flanked by two constant regions. Green fluorescent protein (GFP)-, human heat shock factor 1 (hHSF1)-, and negative elongation factor E (NELF-E)-binding aptamers, GFPapt, HSFapt, and NELFapt, respectively, as well as the background binding sequences (BBSs), BBS1 and BBS2, were all derived from previous multiplex SELEX experiments [13, 15–17]. See Supplementary Material for details.

### RNA selections and quantification

For the sequence-specificity study with serially configured microcolumns, each triplicate of eight targets was exposed to 1 mL of a mixed RNA pool in binding buffer [4.75-nM N70 library, 50-pM GFPapt, 50-pM HSFapt, 50-pM NELFapt, 50-pM BBS1, 50-pM BBS2, and 10-μg/mL yeast tRNA (Invitrogen)]. Similarly for the protein concentration studies with parallel microcolumns, the mixed RNA pools consisted of 4.95-nM N70 library and 50-pM specific aptamer.

After binding to the library, the serially configured microcolumns were reconfigured to run in parallel. Each of the 96 microcolumns was then washed to remove unbound RNA. Finally, MEDUSA was placed directly onto a 2-mL 96-well microplate, and the RNA/RNA-protein complexes were eluted from the individual microcolumns. The RNA elution samples and the input standards were phenol/chloroform-extracted and ethanol-precipitated and the pools and standards were reverse transcribed in two 96-well microplates. Each of the cDNA products was used for quantitative PCR analysis using 384-well plates on a LightCycler 480 instrument (Roche) to determine the amount of RNA library and of each specific aptamer that was recovered from each microcolumn. Different sets of oligonucleotides were used to independently evaluate the amount of N70 library and specific aptamers in each pool. See Supplementary Material for details.

### Design and fabrication of MEDUSA

**Fig. 1:**
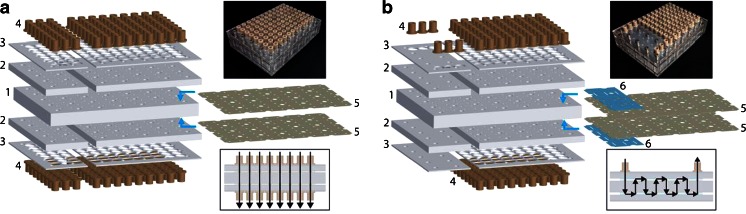
Diagram of the layers of MEDUSA in the order of assembly. (**a**) An exploded view of the customized device layers for configuring all 96 microcolumns to run in parallel. The flow path is shown in the lower boxed inset with no connections between microcolumns. The layers numbered *1* to *3* are the plastic layers: the middle layer (*1*) containing the microcolumns, the next outer two layers being the caps (*2*) and washers (*3*). The outermost layers (*4*) consist of inlet and outlet ports that are bonded to the final device. The two layers numbered (*5*) are silicone layers, which are bonded to the microcolumn layer (*1*) to hold porous frits against both sides of the microcolumns to retain affinity resin and to make liquid-tight seals across the entire device. A photograph of MEDUSA assembled in parallel is shown in the upper inset. (**b**) The customized device layers for configuring 24 of the microcolumns to run in series. The two additional silicone layers (*6*) shown in blue, as well as the smaller complementary plastic layers (*2* and *3*) on the *left*, are specifically programed to connect three sets of eight microcolumns within the device. The flow path is shown in the lower boxed inset with microcolumns connected in series via a serpentine route through eight microcolumns. MEDUSA assembled to run in series and parallel is shown in the *upper inset*

MEDUSA was modeled after a 96-well microplate. The 96 units of our device were based off of our previously reported modular and mutliplexable affinity microcolumns, which were shown to minimize reagent consumption while demonstrating significantly improved performances through optimizations of the selection parameters [13]. In order to allow for simple and versatile multiplexing and connectivity between microcolumns, our device was designed to be assembled in layers, with some of the layers “programed” for establishing connections within the device (see Fig. 1 and Supplementary Material for more details). To fabricate the layers of MEDUSA, a two-dimensional CAD for each layer was designed and then cut using a CO_2_ laser at 10.6 μm (Universal Laser Systems, VersaLaser). The speed, intensity, and density of laser pulses were optimized for each layer to obtain the highest quality and most reproducible cuts.

## Results and discussion

### MEDUSA as an adaptable platform

MEDUSA was designed for high-throughput aptamer selections and characterizations of the SELEX process, and for versatility, allowing any combination of serial and parallel experiments. Due to the availability of plate-based processes and the potential for automation, we designed our device using the standard 96-well microplate layout, which easily couples with a typical 96-well plate for further post-selection sample processing. Furthermore, laser-cutting MEDUSA was ideal for rapid prototyping, requiring only 1 h to machine each device. For a universal device that does not necessitate customized plastic layers, a third layer of silicone could be used to similarly program the accessibility of all 96 possible input and output ports to the microcolumns. However, due to the inexpensive and rapid fabrication methods used, we decided instead to fabricate custom capping and washer layers that relay the same flow program by containing only the necessary input/output holes and NanoPorts. For the three sets of eight serialized microcolumns shown, this required only six holes/ports on the top layers and none on the bottom layers. This configuration also allowed for visual assessment of solutions flowing through the serialized microcolumns. The ease of fabrication for different programed parts, especially in thin silicone, allows for customized and versatile selections that can contain any number of parallel or serially connected microcolumns, as well as utilizing both configurations simultaneously. In cases where more than 96 microcolumns are desired, such as when 96 targets each require negative selections, additional microcolumn layers can be utilized in the assembly. As illustrated, our device was fabricated to accommodate three sets of eight serialized devices, as well as 72 parallel selections (Fig. 1). This combination was easily programed as described above. However, an even greater degree of versatility was achieved by dividing the capping, washer, and programed silicone layers into separately fabricated subsections that could be individually addressed and reconfigured without disrupting other microcolumns. This strategy also suggests the possibility of fabricating smaller versions of MEDUSA that contain the same general layout of a microplate, but occupy a smaller footprint by utilizing fewer microcolumns. This would allow users to handle smaller devices in less demanding applications, while benefiting from the standardized spacing and addressability of plate-based selections and sample processing.

### Parallel selections reveal critical target concentration for aptamer enrichments

In our previous work, we found that GFP aptamer enrichments were limited by a critical GFP concentration that we attributed to steric hindrance [13]. Using MEDUSA, we decided to reproduce the GFP results with more data points, and to investigate the prevalence of this limiting effect by performing analogous studies with two additional proteins, hHSF1 and NELF-E, and their respective aptamers, HSFapt and NELFapt [13, 17]. For each protein target, we chose to test eight concentration conditions starting at 10 μg/μL of resin with 2.5-fold dilutions down to 0.016 μg/μL in triplicate. The layout for all the samples on MEDUSA is illustrated in Fig. S1 in the sections denoted II, III, and IV.

**Fig. 2:**
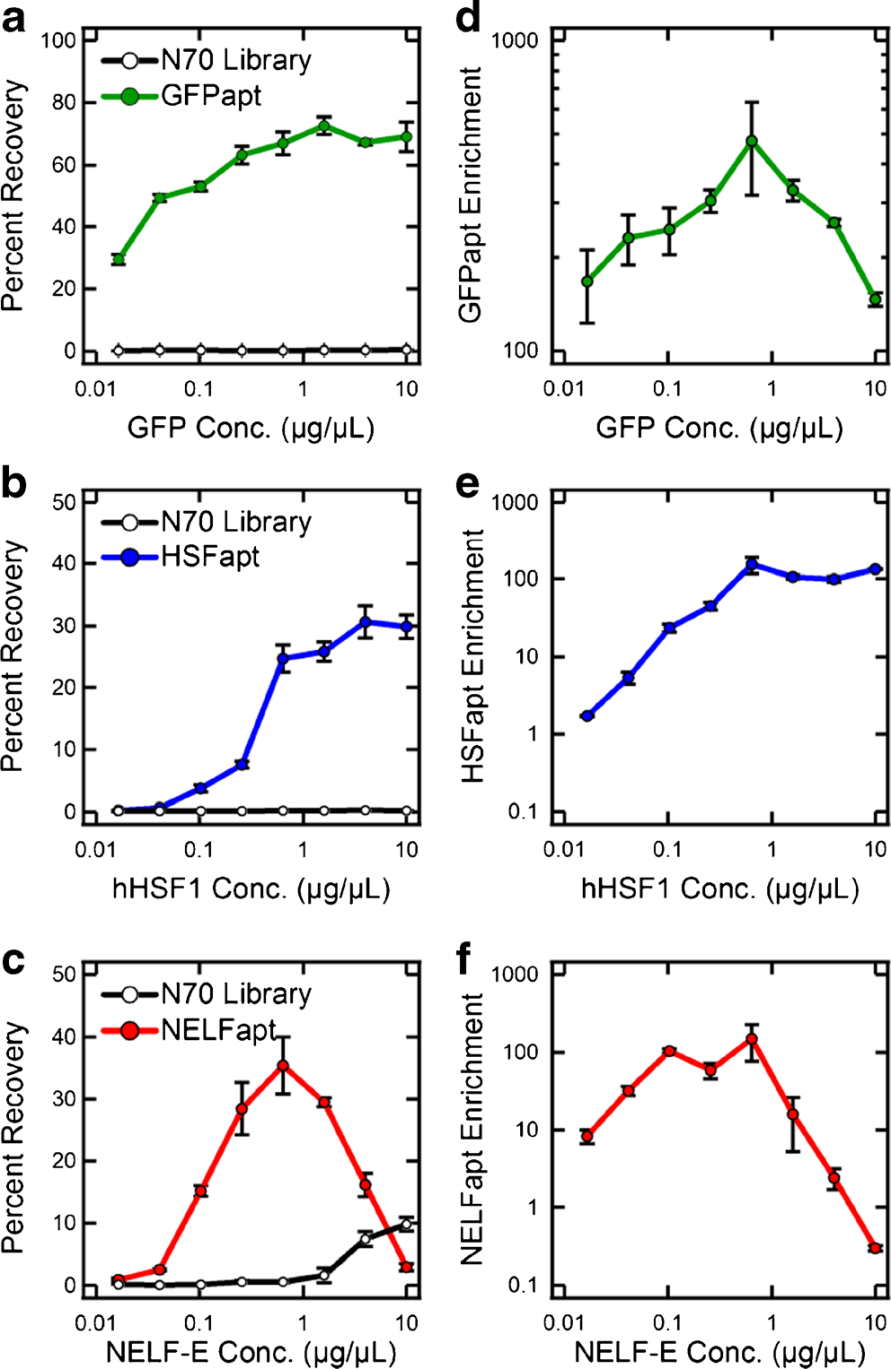
Recoveries and enrichments of specific RNA aptamers over the N70 library as a function of protein concentration. (**a**) The recovery of GFPapt and N70 library at various concentrations of GFP. Analogous data for the recovery of (**b**) HSFapt and N70 library from hHSF1, and (**c**) NELFapt and N70 library from NELF-E. (**d**–**f**) The calculated enrichments of the specific aptamers (GFPapt, HSFapt, NELFapt) over the random library. The *error bars* represent the standard deviation in recoveries or enrichments calculated for each condition performed in triplicate

The binding results and enrichments for all three proteins are shown in Fig. 2. The GFP microcolumns recovered a higher percentage of GFPapt and a lower percentage of N70 library than those reported previously (Fig. 2a), due to lower flow rates that were used to increase HSFapt and NELFapt binding, since they have higher *K*_d_s. This resulted in an expected increase in the enrichment of GFPapt over the N70 library; however, the characteristic shape and optimal concentration of 0.6 μg/μL for the enrichment curve are the same as previously reported (Fig. 2d). With hHSF1, the recovery of HSFapt followed a more typical sigmoidal shape, which saturated at increasing concentrations of hHSF1 (Fig. 2b). Similarly, the enrichment of HSFapt over the N70 library increased steadily and then saturated at higher concentrations (Fig. 2e). It is interesting, however, that HSFapt enrichment plateaued at the optimal concentration for GFP. With NELF-E, there is a very clear NELFapt recovery optimum at this same concentration, with significant losses in recoveries at concentrations above 0.6 μg/μL (Fig. 2c). In addition, the recovery of the N70 library increased significantly above the optimum concentration for NELFapt, likely due to the fact that NELF-E contains an RNA Recognition Motif and can bind RNA non-specifically [17]. These two binding trends result in a drastic decrease in enrichment at higher concentrations of NELF-E, resulting in de-enrichment of NELFapt at the highest concentration of 10 μg/μL (Fig. 2f).

The three concentration studies with GFP, hHSF1, and NELF-E make a strong case for the general steric hindrance of target molecules that are over packed in solid-phase affinity selections. Although the GFP and hHSF1 aptamer recoveries do not show drastic decreases at high concentrations as with NELF-E, this binding behavior is affected by the selection flow rates and is clearly seen between our old and new GFP data. Importantly, the recoveries saturated well below 100 %, which indicates the existence of some limiting effect. Most revealing is NELF-E, where the binding site for NELFapt appears to be particularly inaccessible at high concentrations, causing a significant loss in total aptamer binding. Furthermore, a simple calculation (assuming hard spheres for the resin) predicts that a critical surface density of proteins should occur between 0.1 and 1 μg/μL (depending on protein shape and size and the diameter of the resin beads). Since all three proteins are similar in size, it is not surprising that we observe the same critical concentration of 0.6 μg/μL, and the results suggest that the target concentration may be the most limiting parameter for enriching aptamers.

### Multiplex serial selections show specificity of target and background binding sequences

Previously, we performed multiple partitions to input pools and libraries by connecting several microcolumns in multiplex selections [13]. In particular, we showed the highly specific and efficient partitioning of GFPapt to GFP over non-specific proteins and an empty microcolumn. This configuration is useful for multitasking DNA or RNA libraries on multiple unrelated selection targets, or to separate enriched pools for aptamers that bind to distinct sites on a complex target [18]. We decided to demonstrate similar multiplex selections using MEDUSA by extending this analysis to include several additional RNA aptamers and protein targets: GFP, hHSF1, NELF-E, and their respective aptamers. To thoroughly characterize the specific, non-specific, and background binding of each RNA aptamer, we also included a non-specific protein, UBLCP1, three commonly used affinity resins, GSH, Ni-NTA, and amylose, and empty microcolumns. Each of the four protein targets were immobilized onto their respective resins at 0.6 μg/μL. The eight targets were arranged in series for the multiplex selection and performed in triplicate to quantify the reproducibility of each aptamer’s partitioning efficiency, and specificity. The order of targets was as follows: empty, GSH, Ni-NTA, amylose, His-GFP, GST-hHSF1, His-NELF-E, His-UBLCP1, and is illustrated in Fig. S1 in Section I.

In addition to the random N70 RNA library and the aptamers to our three proteins, our test pool also included two suspected BBSs, BBS1 and BBS2. For all previous multiplex selections, we have performed high-throughput sequencing, which provided tremendous amounts of sequence data and sensitivity for early detection of aptamers [13, 15, 17]. However, comparison of the sequencing results for dozens of targets revealed several identical sequences that were frequently enriched, particularly in earlier cycles before target-binding aptamers began to dominate the pool. This was especially true for less aptagenic targets, where the two sequences, BBS1 and BBS2, were generally among the highest enriched candidates (see Table S2). From these data, we predicted that BBS1 would enrich on all targets by binding to the plastic device and the resins. This was also predicted for BBS2; however, we expected BBS2 to enrich more strongly than BBS1 on all targets, especially in microcolumns containing Ni-NTA (similar analyses have been used to identify sequences that bind specifically to Ni-NTA [19]).

**Fig. 3:**
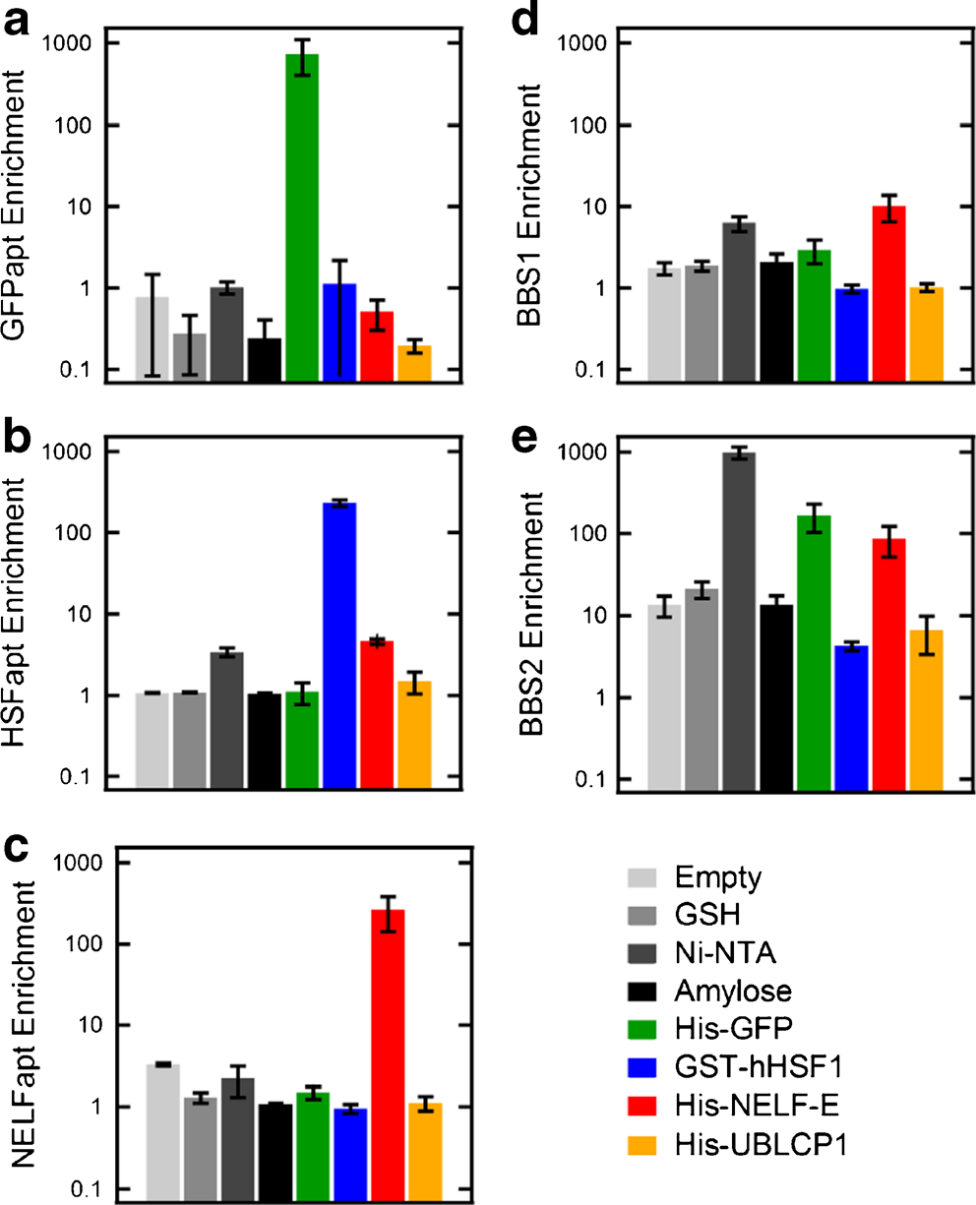
The enrichment of RNA aptamers over the N70 library on various targets connected in series. The enrichment of each protein-specific aptamer, GFPapt (**a**), HSFapt (**b**), and NELFapt (**c**), and non-specific aptamers, BBS1 aptamer (**d**), and BBS2 aptamer (**e**), on all eight microcolumns. The *error bars* represent the standard deviation in enrichments calculated for each target performed in triplicate

The partitioning results for each RNA aptamer are shown in Fig. 3 as enrichments over the random RNA library. Our specific aptamers each show striking enrichments only on their intended target. GFPapt enriched an average of 750-fold on GFP microcolumns, but only an average of 0.6-fold (de-enriched) on all other targets (Fig. 3a), which reflects its strong specificity for GFP. Similarly, HSFapt enriched an average of 232-fold on hHSF1 microcolumns, and only an average of 2-fold on all other targets (Fig. 3b). NELFapt enriched an average of 262-fold on NELF-E, and only an average of 1.6-fold for all other targets (Fig. 3c).

For BBS1 and BBS2, we found good agreement with the qualitative analysis of all previous sequencing data [13, 15, 17]. BBS1 enriched on all targets as predicted; however, it enriched three times more in Ni-NTA-containing microcolumns (Ni-NTA, GFP, NELF-E, UBLCP1), with enrichments averaging 1.7 for non-Ni-NTA targets and 5.1 for the Ni-NTA targets (Fig. 3d). BBS2 also enriched as predicted, with enrichments higher than BBS1 on all targets (Fig. 3e). More specifically, BBS2 enriched an average of 13-fold on non-Ni-NTA targets and a surprising 311-fold on Ni-NTA targets suggesting that BBS2 has a specific affinity for Ni-NTA. In fact, for the first Ni-NTA target in the serial selection, blank Ni-NTA, enrichment averaged almost 1,000-fold. This is almost 80 times greater than non-Ni-NTA targets, and may reflect more accurately the specificity of BBS2 for Ni-NTA. In support of this hypothesis, we noticed that BBS2 was quickly depleted from the pool as it was injected across all the Ni-NTA-containing microcolumns, as seen by the monotonically decreasing enrichments of BBS2 to the Ni-NTA-containing targets from left to right.

Although negative selections are often used to separate sequences with specific affinities for sources of background binding, these are rarely completely effective at eliminating enrichment of non-specific RNAs. In contrast, we have found the repeated occurrence of BBSs in different SELEX experiments to be valuable indicators of the selection progress. Perhaps more interestingly, selections for and/or identification of BBSs can be used to generate non-specific blocking reagents that are more effective than commonly used yeast tRNAs.

## Conclusions

We believe that MEDUSA can be used to significantly increase the productivity of large-scale aptamer discovery efforts. By utilizing the versatility and programmability of MEDUSA, a larger configuration space of potential SELEX designs can be explored. Just as importantly, selections utilizing affinity chromatography can be thoroughly characterized. These kinds of data not only allow us to optimize future aptamer selections, but also clearly show that performances can be improved, while simultaneously consuming much less reagent, such as protein, making aptamer selections more accessible to targets that are difficult to purify or express in large quantities. Furthermore, such characterizations would not only improve future aptamer selections, but also aid in the development of more functional and applicable SELEX theories in solid-phase affinity selections.

## Electronic supplementary material

Below is the link to the electronic supplementary material.

ESM 1 (PDF 610 kb)
