# Supplementary material for: High-throughput binding characterization of RNA aptamer selections using a microplate-based multiplex microcolumn device
Source: Anal Bioanal Chem. 2014 Feb 20;406(11):2727–32. doi: 10.1007/s00216-014-7661-7 (PMC3975076; doi:10.1007/s00216-014-7661-7)
Supplement: Supplementary file 1 — (PDF 610 kb) [file 216_2014_7661_MOESM1_ESM.pdf]

Analytical and Bioanalytical Chemistry

Electronic Supplementary Material

High-throughput binding characterization of RNA aptamer selections using a microplate-based multiplex microcolumn device

Kylan Szeto, Sarah J. Reinholt, Fabiana M. Duarte, John M. Pagano, Abdullah Ozer, Li Yao, John T. Lis, Harold G. Craighead

Preparation of recombinant protein targets

Recombinant proteins were expressed in BL21(DE3)-RIPL *E. coli* cells (Agilent Technologies) transformed with plasmids that encode for hexahistidine-tagged GFP, Drosophila NELF-E, and UBLCP1, or GST-tagged hHSF1 (Table S1). Two or four liter LB cultures supplemented with 100 µg/mL ampicillin were inoculated with starter LB culture derived from a single colony and grown at 37°C until the OD600 reached approximately 0.6. Protein expression was induced by the addition of IPTG to a final concentration of 1 mM. After an additional incubation, bacteria were collected by centrifugation and the resulting pellet was processed according to the manufacturer's instructions for Ni-NTA Superflow (Qiagen) or Glutathione-agarose (Thermo Scientific) resins. SDS-polyacrylamide gel electrophoresis (SDS-PAGE) was used to verify the purity and quality of the final protein product. Resulting protein preps were dialyzed against 1× PBS (supplemented with 5 mM 2-mercaptoethanol and 0.01% Triton X-100) and stored in small aliquots after addition of glycerol to a final concentration of 20%. NELF-E was prepared slightly differently.[1]

Nucleic acid library, protein- and background-binding aptamers

The random N70 library, contains ~5×10^{15} sequences of 120-nucleotide (nt) RNA molecules and was prepared as described previously.[2] This library consists of a 70-nt random region flanked by two constant regions. HSFapt was previously identified as hHSF2-R5-2 using the N70 library and characterized elsewhere.[2] NELFapt was previously identified as Napt1 using the N70 library.[1] The background binding sequences BBS1 and BBS2 were identified in several previous multiplex SELEX experiments using the N70 library for dozens of target proteins.[1-3]

The GFP-binding RNA aptamer, GFPapt, used in this work was selected using a different library with a smaller random region and different constant regions; and was previously identified as AP3-1.[4]

The 84-nt GFP-binding RNA aptamer has the following sequence: 5'-AGCUUCUGGACUGCGAUGGGAGCACGAAACGUCGUGGCGCAAUUGGGUGGGGAAAGUCCUUAAAAGAGGGCCACCACAGAAGCU-3'. The forward and reverse oligos used for qPCR analyses were GFPapt-FOR: 5'-GCTTCTGGACTGCGATGGGAGCA-3' and GFPapt-REV: 5'-GCTTCTGTGGTGGCCCTCTTTTAAGGACT-3'.

The 117-nt hHSF1-binding RNA aptamer has the following sequence: 5'-GGGAAUGGAUCCACAUCUACGAAUUCAAUCAAGUCCCCAGACUCAGCAACACUGGACAGCGAUAUGCAGAUAACCAAGACCAAUUCACUCCAGUUCACUGCAGACUUGACGAAGCUU-3'. The two constant regions corresponding to the library design are denoted by underlines. The forward and reverse oligos used for qPCR analyses were HSFapt-FOR: 5'-AATCAAGTCCCCAGACTCAGCAACA-3' and HSFapt-REV: 5'-CTGGAGTGAATTGGTCTTGGTTATC-3'.

The 120-nt NELF-E-binding RNA aptamer has the following sequence: 5'-GGGAAUGGAUCCACAUCUACGAAUUCCCAACGACUGCCGAGCGAGAUUACGCUUGAGCGCCCCACUGAGGAUGCCCACGGGCGAUUGGGGCACGGCUUCACUGCAGACUUGACGAAGCUU-3' The two constant regions corresponding to the library design are denoted by underlines. The forward and reverse oligos used for qPCR analyses were NELFapt-FOR: 5'-CCAACGACTGCCGAGCGAGATTAC-3' and NELFapt-REV: 5'-GCCGTGCCCCAATCGCCCGTG-3'.

BBS1 has the following sequence: 5'-GGGAAUGGAUCCACAUCUACGAAUUCCGCAGGGCUAGCCGCAUGCUCAGGCCUGGCGGGUAGGGAGUUAGGGUAGGGAGACCAGGAGAGCUGGCUUCACUGCAGACUUGACGAAGCUU-3'. The forward and reverse oligos used for qPCR analyses were BBS1-FOR: 5'-CGCAGGGCTAGCCGCATG-3' and BBS1-REV: 5'-GCCAGCTCTCCTGGTCTCC-3'.

BBS2 has the following sequence: 5'-GGGAAUGGAUCCACAUCUACGAAUUCCGAAGCUCGUGACGGUACCUCCUAAAAUGUCCAUGGGGAAGGGAGGGAAUGGGAAGGACAAUCGGACACCGUUCACUGCAGACUUGACGAAGCUU-3'. The forward and reverse oligos used for qPCR analyses were BBS2-FOR: 5'-CGAAGCTCGTGACGGTACC-3' and BBS2-REV: 5'-CGGTGTCCGATTGTCCTTC-3'.

The N70 library forward and reverse oligos used for qPCR analyses were Lib-FOR oligo: 5'-GATAATACGACTCACTATAGGGAATGGATCCACATCTACGA-3' and Lib-REV oligo: 5'-AAGCTTCGTCAAGTCTGCAGTGAA-3'.

All of the oligos used in this work were obtained from Integrated DNA Technologies.

Preparation of protein- and background-binding aptamers

Sequence verified DNA templates for each one of the specific aptamers used in this study were transcribed using T7 RNA Polymerase. After transcription, the samples were treated with DNase I (Ambion), PAGE-purified, phenol:chloroform and chloroform extracted, isopropanol precipitated, and then re-suspended in DEPC-treated H_2O.

Table S1: Properties of the target proteins

Protein	Molecular Weight (kDa)	Isoelectric Point	Affinity tag
GFP	27	5.5	Hexahistidine (n-terminus)
hHSF1	86	5.3	GST* (n-terminus)
NELF-E	36	8.9	Hexahistidine (n-terminus)
UBLCP1	37	6.1	Hexahistidine (c-terminus)

*GST tag ~ 30 kDa

RNA selections and quantification

The RNA pools were injected at a rate of 33 µL/min for 30 min with a 10 µL aliquot of each pool set aside and used as a standard for quantitative polymerase chain reaction (qPCR) analysis. All buffers and solutions were degassed prior to use and introduced into the microcolumns via programmable multichannel syringe pumps (Harvard Apparatus) with MEDUSA placed onto a 96-well format liquid waste reservoir. The microcolumns were reconfigured to run in parallel by removing the caps and silicone layers permitting the connectivity of microcolumns, and reassembling the device with the appropriate caps for a parallel configuration, and washed with 3 mL of binding buffer at a rate of 300 µL/min. The RNA/RNA-protein complexes were eluted directly into a 96-well microplate from the individual microcolumns by flowing elution buffer [binding buffer + 50 mM ethylenediaminetetraacetic acid (EDTA pH 8.0) for selections with Ni-NTA resin; binding buffer + 10 mM glutathione for selections with GSH resin; binding buffer + 10 mM maltose for selections with amylose-resin] at a rate of 50 µL/min for 12 min. Samples and standards were phenol/chloroform-extracted and ethanol-precipitated together with 1 µL of GlycoBlue (Ambion) and 40 µg of yeast tRNA (Invitrogen), and the resulting pellet was resuspended in 20 µL of RNase-free water, and reverse transcribed with Moloney Murine Leukemia Virus Reverse Transcriptase (MMLV-RT) in two 96-well microplates. The N70 library, HSFapt, NELFapt, BBS1, and BBS2 all contain the same 3' constant region and were reverse transcribed using Lib-REV primer complementary to the 3' constant region in the RNA. For the experiments containing GFPapt, 4 µL of the resuspended pools and the standards were reverse transcribed using the GFPapt-REV primer specific to GFPapt. A 10-µL volume of each of the cDNA products was used for qPCR analysis using 384-well plates on a LightCycler 480 instrument (Roche) to determine the amount of RNA library and of each specific aptamer that was recovered from each microcolumn. Different sets of oligonucleotides (see above) were used to independently evaluate the amount of N70 library and specific aptamers in each pool.

Descriptions of MEDUSA's Components

Each layer of MEDUSA was fabricated from either transparent biocompatible poly(methyl methacrylate) (PMMA) plastic or silicone. As seen in Figure 1A (lower boxed inset), for parallelized microcolumns, there are 5 layers of plastic and 2 layers of silicone as well as NanoPorts (IDEX Health and Science) for inputs and outputs on each side. The center most plastic layer (number "1" in Figure 1A) is 1/2" thick and contains 96 microcolumns that each hold 10 μL of total volume. The next pair of layers (numbered "5" in Figure 1A above and below the microcolumns) are 1/16" silicone layers for making a liquid tight seal across all 96 microcolumns. These layers contain 2 mm diameter holes for inserting porous polyethylene frits above and below each microcolumn to retain target-bound affinity resins, and have adhesive on one side for bonding to the microcolumn layer. The next pair of layers (numbered "2" in Figure 1A) are 1/4" plastic capping layers which have small holes and NanoPorts (numbered "4") bonded around them to allow solutions to flow in and out of the microcolumns. The outer most plastic layers (numbered "3"in Figure 1A) are 1 mm thick and designed to simultaneously aid the alignment of the NanoPorts to the capping layers, as well as to bear and distribute forces from the assembly of all the layers by acting as a washer. All of the layers contain 35 evenly-spaced holes, with the middle microcolumn layer being threaded, for sealing the device together with screws (Figure 1A and 1B upper inset photographs). For serialized microcolumns (Figure 1B, lower boxed inset), the design and assembly is similar. However, there are 2 additional layers of silicone (numbered "6" in Figure 1B). These layers are fabricated in 1/32" silicone (no adhesive) and are programed to allow for the connectivity of microcolumns through small interconnecting channels.

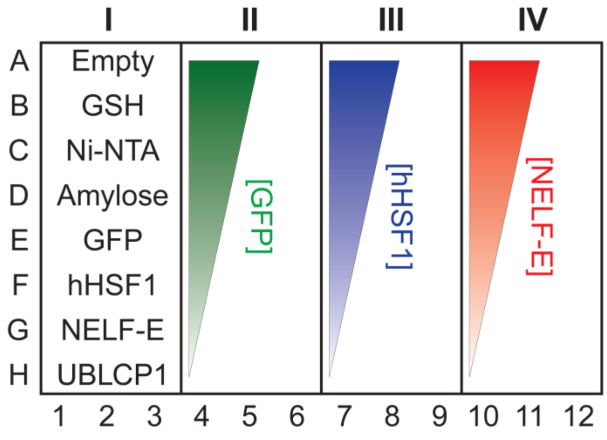

Fig. S1 Layout for the 96 targets on MEDUSA according to its analogous microplate position given by the rows A-H, and the columns 1-12. In section I, the 8 indicated targets were connected in series from A to H to test the specificity and partitioning efficiency of various RNA aptamers. This was tested in triplicate in columns 1 to 3. Sections II, III, and IV tested the effects of target surface concentration on aptamer enrichments. The colored triangles indicate decreasing concentrations of each protein from 10 μg/μL (row A) to 0.016 μg/μL (row H) in 2.5-fold dilutions. Section II (green triangle) aimed to confirm previous enrichment behaviors shown with GFP. Sections III and IV tested the same concentrations of the proteins hHSF1 (blue triangle) and NELF-E (red triangle) to assess the prevalence of target surface concentration effects on binding due to steric hindrance or other effects in other aptamer selections

<u>Table S2: Frequencies of BBS1 and BBS2 in previous selections.</u> Summary of the number of times BBS1 or BBS2 has been identified in all previous selections. The numbers indicate the instances in which BBS1 was more highly enriched than BBS2 (or vice versa) on each target, grouped according to the resin on which each target was immobilized.

Resin	BBS1 Dominant	BBS2 Dominant
Ni-NTA	1	7
GSH	3	4
Amylose	3	4
Empty (no Resin)	0	2

References

1. Pagano JM, Kwak H, Waters CT, Sprouse RO, White BS, Ozer A, Szeto K, Shalloway D, Craighead HG, Lis JT (2014) Defining NELF-E RNA Binding in HIV-1 and Promoter-Proximal Pause Regions. PLoS Genetics 10:e1004090.
2. Latulippe DR, Szeto K, Ozer A, Duarte FM, Kelly CV, Pagano JM, White BS, Shalloway D, Lis JT, Craighead HG (2013) Multiplexed microcolumn-based process for efficient selection of RNA aptamers. Analytical chemistry 85:3417-3424.
3. Szeto K, Latulippe DR, Ozer A, Pagano JM, White BS, Shalloway D, Lis JT, Craighead HG (2013) RAPID-SELEX for RNA Aptamers. PloS one 8:e82667.
4. Shui B, Ozer A, Zipfel W, Sahu N, Singh A, Lis JT, Shi H, Kotlikoff MI (2012) RNA aptamers that functionally interact with green fluorescent protein and its derivatives. Nucleic Acids Res 40:e39.
